# NMF-mGPU: non-negative matrix factorization on multi-GPU systems

**DOI:** 10.1186/s12859-015-0485-4

**Published:** 2015-02-13

**Authors:** Edgardo Mejía-Roa, Daniel Tabas-Madrid, Javier Setoain, Carlos García, Francisco Tirado, Alberto Pascual-Montano

**Affiliations:** 1 ArTeCS Group, Department of Computer Architecture, Complutense University of Madrid (UCM), Madrid, 28040 Spain; 2 Functional Bioinformatics Group, Biocomputing Unit, National Center for Biotechnology-CSIC, UAM, Madrid, 28049 Spain

**Keywords:** Non-negative Matrix Factorization (NMF), Graphics-Processing Unit (GPU), CUDA, Multi-GPU implementation, Message Passing Interface (MPI), Biclustering analysis, Sample classification, Gene-expression analysis

## Abstract

**Background:**

In the last few years, the ***Non-negative Matrix Factorization*** **(** ***NMF*** **)** technique has gained a great interest among the Bioinformatics community, since it is able to extract interpretable parts from high-dimensional datasets. However, the computing time required to process large data matrices may become impractical, even for a parallel application running on a multiprocessors cluster.

In this paper, we present ***NMF-mGPU***, an efficient and easy-to-use implementation of the NMF algorithm that takes advantage of the high computing performance delivered by ***Graphics-Processing Units*** **(** ***GPUs*** **)**. Driven by the ever-growing demands from the video-games industry, graphics cards usually provided in PCs and laptops have evolved from simple graphics-drawing platforms into high-performance programmable systems that can be used as coprocessors for linear-algebra operations. However, these devices may have a limited amount of on-board memory, which is not considered by other NMF implementations on GPU.

**Results:**

*NMF-mGPU* is based on ***CUDA*** **(** ***Compute Unified Device Architecture*** **)**, the NVIDIA’s framework for GPU computing. On devices with low memory available, large input matrices are blockwise transferred from the system’s main memory to the GPU’s memory, and processed accordingly. In addition, *NMF-mGPU* has been explicitly optimized for the different CUDA architectures. Finally, platforms with multiple GPUs can be synchronized through ***MPI*** **(** ***Message Passing Interface*** **)**. In a four-GPU system, this implementation is about 120 times faster than a single conventional processor, and more than four times faster than a single GPU device (i.e., a *super-linear* speedup).

**Conclusions:**

Applications of GPUs in Bioinformatics are getting more and more attention due to their outstanding performance when compared to traditional processors. In addition, their relatively low price represents a highly cost-effective alternative to conventional clusters. In life sciences, this results in an excellent opportunity to facilitate the daily work of bioinformaticians that are trying to extract biological meaning out of hundreds of gigabytes of experimental information. *NMF-mGPU* can be used “*out of the box*” by researchers with little or no expertise in GPU programming in a variety of platforms, such as PCs, laptops, or high-end GPU clusters. *NMF-mGPU* is freely available at https://github.com/bioinfo-cnb/bionmf-gpu.

## Background

The recent advances in the high-throughput technologies used in Biology generate large amounts of data that require analysis and interpretation. Multiple data-mining methods are very useful for hypothesis formulation and exploratory analysis of biological datasets. Clustering algorithms or matrix factorization techniques, such as ***Principal Component Analysis*** **(** ***PCA*** **)** [1], ***Singular Value Decomposition*** **(** ***SVD*** **)** [2], or ***Independent Component Analysis*** **(** ***ICA*** **)** [3], are among the most popular tools to find natural group structures in high-dimensional datasets.

The ***Non-negative Matrix Factorization*** **(** ***NMF*** **)** [4,5] technique has also been established as a very effective method to discover biological patterns. NMF decomposes a large input dataset into a small set of highly interpretable and additive parts. This property has centered the attention of scientists for a wide range of applications in Bioinformatics, such as gene-expression analysis [6,7], scientific literature mining [8], neuroscience [9], and several *-omics* techniques [10,11]. A good review of some of them can be found in [12]. Other fields of science also use NMF. For example, face and object recognition [5,13], color science [14], computer vision [15], polyphonic music transcription [16], as well as other signal-processing methods [17,18].

The interest on this technique by the bioinformatics community has yield to several standalone applications [19], online tools [20-22], and code in different programming languages [23-25]. The scientific community has also reported some conventional parallel implementations intended for other fields of science [15,26]. However, the usage of any of these applications is hindered by the large and constantly growing datasets that require analysis, especially in fields like Genomics or Proteomics. For instance, since the release in June 2008 of our public web tool, ***bioNMF*** [20], the server has registered an average of 75 jobs per month. In spite of using a parallel implementation of NMF in an eight-processors system, some of these jobs took tens of hours to complete, monopolizing the cluster and increasing the response time of subsequent submissions.

Even on a dedicated local cluster, the required computing time may become impractical in many scenarios. An example is the application of NMF to the exploratory data analysis, which usually involves numerous executions of the NMF algorithm using different parameters (e.g., [23]). Another scenario results from the development of high-throughput sequencing technologies and the potential bottleneck caused by NMF, since experimental data may be generated at a higher rate than it can be analyzed. Therefore, a new strategy to improve the performance of the NMF algorithm is highly desirable.

In this paper, we present an alternative implementation of the NMF algorithm based on a programmable ***Graphics-Processing Unit*** **(** ***GPU*** **)**. GPUs are devices specially designed to perform the numerous linear-algebra operations required to draw graphics on the screen much faster than any conventional processor (CPU: Central Processing Unit) [27]. The high-performance parallel architecture of GPUs has evolved to a general-purpose programmable system that, working as a coprocessor, is able to execute non-graphics-related applications [28].

All these features have centered the attention of scientist in a wide range of fields, such as image processing [29], molecular dynamics, quantum chemistry [30], and physics [31], among many others. There are, in addition, other publications focused on different approaches to perform particular linear-algebra operations (e.g., matrix multiplications [32] and LU Factorizations [33]), as well as generic linear-algebra libraries [34] and plug-ins for other systems [35]. In Bioinformatics, GPUs have been used, for instance, in gene-expression connectivity mapping [36], sequence alignment [37], and protein docking [38], among others [39].

Nevertheless, most GPU architectures are presented in the form of a detached device connected to the CPU via a system bus (e.g., PCI Express), and equipped with a dedicated on-board memory. This configuration requires the transfer of the input data from the CPU’s main memory to the GPU’s memory before starting the process. Similarly, after the computing process is finished, the output data must be transferred back to the CPU’s memory in order to retrieve the results.

Moreover, the amount of such on-board memory ranges from hundreds of megabytes to a few gigabytes, which is usually much less than its CPU counterpart. Therefore, in order to analyze large datasets, the algorithm must be able to explicitly blockwise transfer and process the input data. This sequential procedure must be performed with care to avoid severe negative effects on performance.

To the best of our knowledge, there are only a few GPU implementations of the NMF algorithm [16,40-42], but these domain-specific applications do not perform any blockwise processing since they do not consider the available amount of GPU memory, nor make use of multiple GPU devices. Therefore, they are not suitable for the analysis of current large biological datasets.

Conversely, *NMF-mGPU* is an application able to process datasets of any size using a single or multiple GPU devices. An in-depth performance analysis of a preliminary version of this application can be found in [43]. It shows that negative consequences of blockwise processing can be mitigated with the use of multiple GPUs, or a multi-GPU system, where multiple data blocks can be simultaneously transferred and processed. Nevertheless, attention must be paid to avoid excessively increase the number of devices, and thus, the overhead due to inter-device synchronizations [43]. In this work, our goal is not only to show the outperformance of GPU implementation over conventional CPU processing, but also to provide an easy-to-use NMF software package that, “*out-of-the-box*”, can be used in a laptop by any researcher with little or no expertise in GPU programming, or by experimented people in a high-end multi-GPU system.

## Implementation

### The Non-Negative Matrix Factorization (NMF)

One of the most popular applications of NMF in Bioinformatics is the ***Gene-expression Analysis*** [6,7]. It is based on the ***Microarray Technology***, which is a powerful method able to monitor the expression level of thousands of genes, or even whole genomes, in a single experiment [44]. The generated information is usually stored in a numerical matrix whose rows represent genes in a given organism, and the columns correspond to different experimental conditions or samples. Therefore, cells in this matrix encode the relative abundance, or ***expression level***, of a given gene in a certain experimental condition [45].

**Figure 1:**
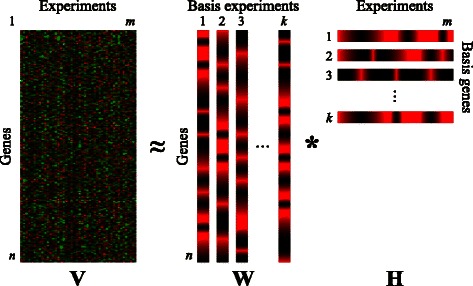
**Schematic representation of the NMF model applied to gene-expression data.** Input matrix **V** represents a gene-experiment matrix to be decomposed as the product of two matrices, **W** and **H**, encoding, respectively, the most significant genes, and experimental samples.

NMF is able to transform this matrix into the linear combination of a reduced set of elements named ***factors***. The usually low number of such components reduces considerably the dimensions of data to be analyzed. Mathematically, NMF can be described as the decomposition of an input matrix (**V** ∈ R^*n* × *m*^) into two other matrices (**W** ∈ R^*n* × *k*^ and **H** ∈ R^*k* × *m*^, where *k* < < *n, m*) constrained to have non-negative elements, and whose product is approximately the former (i.e., **V** ≈ **W** * **H**). In this way, **W** contains the reduced set of *k* factors, and **H** stores the coefficients of the linear combination of such factors that rebuilds **V**. Note that the number of factors, *k*, is generally chosen to a value much less than *n* and *m*. Figure 1 shows a graphic representation of the model when used on gene-expression data.

NMF iteratively modifies **W** and **H** until their product approximates to **V**. Such modifications, composed by matrix products and other algebraic operations, are derived from minimizing a cost function that quantifies in some way the differences between W*H and **V**. There are numerous objective functions, leading each to different update rules [4,46]. Similar to our both previous releases of *bioNMF* [19,20], the new *NMF-mGPU* implements the following rules taken from [23]:

$$ \begin{array}{l}{H}_{pj}\kern1em \leftarrow \kern1em {H}_{pj}\frac{{\displaystyle \sum_{i=1}^n{W}_{ip}{V}_{ij}/{(WH)}_{ij}}}{{\displaystyle \sum_{r=1}^n{W}_{rp}}}\\ {}{W}_{ip}\kern1em \leftarrow \kern1em {W}_{ip}\frac{{\displaystyle \sum_{j=1}^m{V}_{ij}{H}_{pj}/{(WH)}_{ij}}}{{\displaystyle \sum_{t=1}^m{H}_{pt}}}\end{array} $$

In contrast to other similar factorization algorithms (e.g., PCA [1], SVD [2], or ICA [3]), NMF is constrained to use non-negative values and additive-only combinations on all matrices. This results in a parts-based representation of data, where each *factor* can be interpreted contextually [5].

In the last few years, some variants of this algorithm have been proposed in order to enforce sparseness on the resulting matrices [47,48], since there is no explicit guarantee in the method—other than the non-negativity constraints—to support a parts-based representation of the data [49]. Others variants try to increase the effectiveness and the speed of the algorithm on biological data by relaxing such non-negativity constraint, as well as the alternate application of the update rules (i.e., one of the output matrices may be updated a few times while the other stays fixed, and vice versa) [50].

With the development of different expression-profiling techniques [51], a large collection of gene-expression datasets has been made available to the scientific community. They constitute reference databases or *“compendiums”* of gene-expression profiles in the study of a variety of biological systems [52-54]. Many of these databases may contain the expression level of entire genomes analyzed on thousands of experimental conditions. Therefore, they are ideal candidates to be used as input for NMF and similar algorithms.

One of the most popular applications of NMF in gene-expression analysis is ***Biclustering Analysis*** [55,56]. It is a two-way clustering method that identifies groups of genes and experimental conditions that exhibit similar expression patterns. This results in sets of genes similarly expressed in subsets of experimental conditions. Identification of such block-structures plays a key role to get insights into the biological mechanisms associated to different physiological states, as well as to define gene-expression signatures [55,56].

Another popular analysis method is ***Sample Classification*** [23]. This method makes use of NMF and a model-selection algorithm to determine the most suitable number of clusters into which samples (or experiments) can be grouped. This model, based on a reduced set of metagenes, provides a more accurate and robust classification than other methods based on the high-dimensional gene space [23]. Nevertheless, since this method is based on repeating the NMF algorithm numerous times (most typically, in the order of several hundred of times) using different parameters, it is crucial to have an efficient implementation of NMF.

### The CUDA programming model

We have developed our GPU implementation of the NMF algorithm taking advantage of NVIDIA’s GPU programming framework: ***CUDA*** (***Compute Unified Device Architecture***) [57]. CUDA presents GPUs as systems composed by hundreds or thousands of very simple processors—named ***cores***—that allow the simultaneous execution of a given instruction on different data. For instance, when adding a scalar value to a vector, all cores work simultaneously, each performing the addition to a different vector component. This feature makes GPUs very convenient for linear-algebra algorithms which can be processed much faster than on any single-processor system [27].

**Figure 2:**
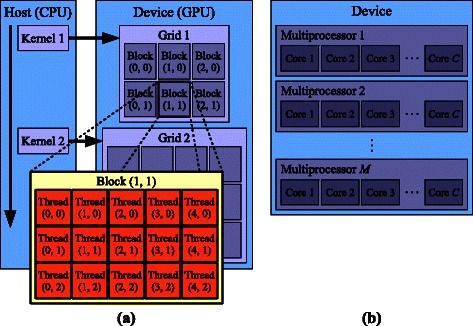
**CUDA (a) hardware and (b) software entities hierarchy.**

The GPU cores are grouped into several multiprocessors integrated in the same GPU (see Figure 2a), being their distribution and number specific to each GPU architecture. The execution of instructions in a CUDA program is carried out by CUDA threads. As shown on Figure 2b, these threads are organized into a grid of ***CUDA blocks***. Within certain constraints, the programmer can specify the number of blocks and threads per block to be executed. At runtime, the GPU executes the threads according to its physical computing resources, so that devices with a larger number of cores will simultaneously execute more threads than devices with fewer processors. The programming model is closely related to this organization: each CUDA block is executed by one multiprocessor chip, which depending on the resource availability can accommodate multiple blocks concurrently. The scheduling unit, named ***warp***, is a set of 32 threads. Every two clock cycles, the scheduler in each multiprocessor chooses the next warp of threads and executes them in a ***SIMT*** (***Single-Instruction Multiple-Threads***) fashion, one half-warp at a cycle. In this model, the programmer implements the generic behavior of a single thread during all the computing process. Such code is named ***CUDA Kernel***. Then, at runtime, the scheduler selects the next single instruction from the kernel and executes it for the entire warp.

To help programmers to implement new applications or migrate existing code to GPU Computing, CUDA provides an extension to the ***C*** language to (i) allocate memory on the GPU, (ii) transfer data between the CPU’s main memory and the GPU’s memory, and (iii) launch *kernels* on the GPU. CUDA also provides inter-thread communication and synchronization mechanisms. Threads within a block can cooperate with each other by (i) sharing data through a fast—but low-capacity—shared memory, and (ii) synchronizing their execution via thread barriers. In contrast, threads from different blocks can only communicate with each other via a slower—but much higher-capacity—global memory. However, there is no mechanism to synchronize the execution of threads in different blocks, so extra care must be taken in order to avoid race conditions. Both resources constitute the GPU’s on-board memory, and a proper management of such is critical to the performance of algorithms. For instance, accesses to shared memory are a hundred times faster than accesses to global memory.

Finally, CUDA classifies the existing GPU architectures with the term ***“Compute Capability X.Y”***, where “*X”* denotes the GPU generation step, and “*Y”* quantifies the evolution level within that generation. For instance, *“Compute Capability 3.5”* represents a medium evolution within the third generation of GPU devices.

### NMF on Graphics-Processing Units (GPUs)

As described in the Background section, a critical resource in this work is the limited size of the on-board GPU memory, which forces to design a data-decomposition scheme in order to process large datasets.

**Figure 3:**
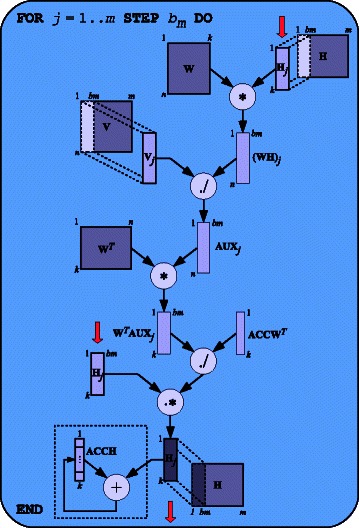
**Update rule for matrix H.** Matrix **V** is blockwise transferred, while **W** and **H** are fully loaded into the GPU memory at algorithm start. Nevertheless, both **V** and **H** are processed in portions of size n × *b*_*m*_ and *k* × *b*_*m*_, respectively (*b*_*m*_ ≤ *m*). Circled operations denote CUDA Kernels. Symbols *“* .* *”* and *“* ***./*** *”* denote pointwise matrix operations. Updated columns from **H** are marked with a big down arrow. Finally, the squared region at bottom left represents the reduction and accumulation of updated columns into a single *k*-length vector required by the next update-**W** rule.

Figure 3 shows the update-rule scheme for matrix **H**. The main loop processes on each iteration, *b*_*m*_ columns from **H** (*b*_*m*_ ≤ *m*). In order to update such columns, it requires a full copy of **W** and the corresponding *b*_*m*_ columns from **V**. Matrix products are computed by the NVIDIA’s ***CUBLAS Library*** [34], while the rest of operations are performed by our CUDA kernels. Since the next update-**W** rule requires all columns from **H** to be reduced to a single *k*-length vector (see denominator in second equation of the NMF algorithm), we take advantage of the temporal locality in the update-**H** rule by reducing and accumulating each set of *b*_*m*_ columns once they are updated. This process is represented in the squared region at bottom left of Figure 3. The reduction kernel allocates memory for a *k*-length vector and performs an inner iterative process. The update rule for matrix **W** is similar. In this case, the loop iterates over *b*_*n*_ rows (*b*_*n*_ ≤ *n*) from matrices **W** and **V**. Note that both **W** and **H** are always fully loaded into the GPU memory since *k* is usually low. This simplifies the implementation and reduces data transfers.

In addition to the data-decomposition scheme, a proper geometry of CUDA blocks and their distribution into a grid must be decided for all kernels we have developed. For this implementation, a configuration of 1D blocks in a 1D grid seems to provide the best performance. Since data matrices are stored in memory as large vectors, successive CUDA threads can be mapped to contiguous matrix data. This ensures that threads in the same warp access to consecutive addresses in global memory. Proper data alignment in memory is also ensured by forcing all matrix widths and vector sizes to be a multiple of the warp size. Other minor architecture-specific optimizations, mostly related to arithmetic instructions and data sharing among threads within a warp, may be also enabled at compile time according to the selected target GPU device(s).

Data transfers are performed asynchronously, so they can be overlapped with kernel executions. Modern GPU architectures can also execute different kernels simultaneously. In both cases, concurrency is managed by ***CUDA Streams*** and ***CUDA Events***.

Finally, it is important to mention that matrix **H** is internally stored in memory with column-major order (i.e., it is transposed), so that **W** and **H** have the same *“width”* (i.e., *k “columns”*) and kernels can be reused on both update rules.

### NMF on Multi-GPU devices

*NMF-mGPU* can operate on multiple GPU devices synchronized through ***MPI*** (***Message Passing Interface***) to provide even more parallelism, especially on very large datasets. In this case, the input matrix is distributed among the existing devices and each portion is processed as described for a single device. That is, all the iterations of the loop shown in Figure 3 are then distributed and simultaneously executed on multiple devices. Since each GPU has to manage a smaller problem, the conjunction of both levels of parallelism also represents a reduction of data transfers between the CPU main memory and the GPU memory. Furthermore, if such data portions are small enough, they can be transferred only once at algorithm start.

Nevertheless, this hybrid version also imposes new overheads. Since each device requires a full copy of both matrices **W** and **H**, a collective-communication step is necessary after performing each update rule in order to keep coherence among all local replicas. Furthermore, such communication steps must be performed through the CPU, so that each device must previously transfer the updated data to the main memory. Similarly, the resulting synchronized data must be transferred back to the on-board memory.

## Results and discussion

### Performance

Our experimental evaluations have been performed on an Intel workstation equipped with four NVIDIA Tesla C1060 GPUs connected via a PCI Express bus. Each device has 4 GB of memory, a Compute Capability of 1.3, and 240 cores distributed on 30 multiprocessors. Our software was compiled and tested on a 64-bits Linux with gcc 4.4.5, CUDA 3.2, and mpich 1.2.7p1. For performance reasons, all arithmetic operations on floating-point data were executed in single-precision mode; however, *NMF-mGPU* can be easily configured to make use of double-precision data on capable devices. Finally, it is important to mention that, although our tests were performed on old GPU devices with an also old CUDA release, all results are still valid for newer hardware/software systems. That is, as stated on the previous section, *NMF-mGPU* has been explicitly optimized for the different generations of CUDA-capable devices. This guarantees that it is widely available to almost anyone, which was one of our central goals in this proposal.

Three gene-expression datasets were tested. They represent small-, medium-, and large-size classes, respectively:

- ***ALL-AML (5000 × 38):*** A set of 5000 genes analyzed from 38 bone marrow samples, corresponding to two tumoral tissues: *Acute Lymphoblastic Leukemia* (*ALL*) and *Acute Myelogenous Leukemia* (*AML*) [58].
- ***ExpO (54675 × 1973):*** A set of 1973 tumor samples obtained by the expO project [53], which are available at Gene Expression Omnibus [52] (accession number: GSE2109).
- ***HG (22283 × 5896):*** Microarray data from almost 6000 human samples representing different cell and tissue types, disease states and cell lines. They were collected by [59] and made available at ArrayExpress [54] (accession number: E-TABM-185).

All tests were performed for different factorization ranks (*k*), ranging from 2 to 10 factors. In order to have the closest test conditions, matrices **W** and **H** were initialized with “random” values generated from the same seed. In addition, all tests correspond to only the first 440 iterations of the NMF algorithm, preventing the convergence criterion to affect the computing time. Our baseline sequential code used for reference, was executed on an IBM PowerPC 970 FX (2.2 GHz, 4 GB of memory), compiled with XLC, and linked with the ATLAS library v3.6 [60] for faster matrix operations.

It is worth to mention that our tests do not to include a performance comparison between *NMF-mGPU* and the other NMF implementations on GPU [16,40-42] described in the Background section. As previously stated, these applications do not take into account the available GPU memory, nor make use of multiple GPU devices. Therefore, only small-or medium-size datasets could be tested. In this reduced scenario, *NMF-mGPU* is clearly in disadvantage, since its capability of blockwise processing large datasets always entails a slightly inherent overhead (e.g., management of different CUDA streams and events, data transfers, block sizes, etc.), even in the analysis of a small input matrix. Furthermore, as test results show below, this overhead becomes noticeable in the multi-GPU version.

### Performance of the single-GPU version

Although the 4 GB of memory available on the GPU device is enough to fully load any of the three datasets, our tests were performed by limiting that amount of memory to 800 MB. This forced the largest dataset (“*HG*”) to be blockwise transferred from the CPU to the GPU, so we could measure the impact of such data transfers.

**Figure 4:**
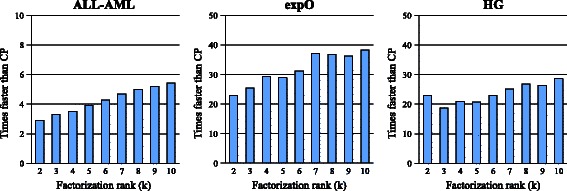
**Speedups of a single GPU compared to a CPU.**

Figure 4 illustrates the speedup of a singe GPU compared with CPU execution times. On the smallest dataset (“*ALL-AML*”), speedups are modest (less than 6x compared to the CPU), since there is not enough useful work to sustain thousands of threads running concurrently. On the medium-size dataset (“*ExpO*”), which fit into the GPU memory, it performs up to 38 times faster than the CPU. Finally, on our largest data set (“*HG*”), the speedup is reduced to 29× due to the overhead of frequent data transfers, which represent about 30% of the time.

### Performance of the Multi-GPU version

**Figure 5:**
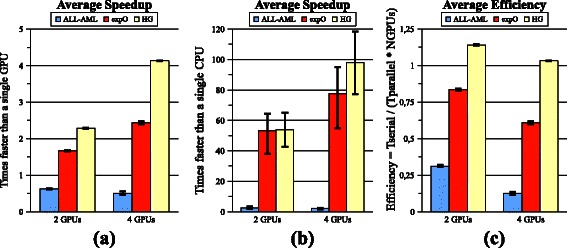
***NMF-mGPU*** **performance.** Average speedups of the multi-GPU version compared to **(a)** a single GPU, and **(b)** to a conventional CPU. **(c)** Efficiency achieved compared to a single GPU. It is defined as the speedup divided by the number of GPUs.

Figures 5a and 5b show the average speedups compared to a single GPU and to a single CPU, respectively. In addition, the efficiency of this hybrid implementation is depicted in Figure 5c. The Efficiency quantifies the utilization of each GPU. A value of 1.0 denotes that synchronization overheads do not affect the performance.

Compared to a single GPU device (Figure 5a), results show a worst or similar pattern on both small and medium-size datasets, respectively, due to the increased number of data transfers and synchronization overheads. In contrast, on the “*HG*” dataset, it achieves a ***super-linear speedup*** *.* That is, with two (four) GPUs, it performs faster than twice (four times) the speed of a single device. This is possible since the portion of the input matrix distributed to each device is small enough to be transferred only once, at algorithm start. This largely compensates all other synchronization overheads.

### Software usage

*NMF-mGPU* consists of two executable files, one to execute the algorithm in a single GPU, and the other to operate multiple devices through MPI. The latter can also be used in a single device with a little overhead produced by the MPI synchronization mechanism. In the project webpage (https://github.com/bioinfo-cnb/bionmf-gpu), we included a short tutorial on how to install the package and its dependencies.

We have implemented an easy-to-use and simple executable code that allows a researcher with no experience to use the advantages of these devices. For the single-GPU case, the software consists on one binary file, “NMF_GPU”, which performs the standard NMF analysis in a single GPU device. Valid input-file formats are the same as our previous web tool, *bioNMF* [20], including ASCII text files with tab-separated data, and binary files encoded using IEEE little-endian byte ordering. The latter is a standard format widely used in many applications.

Input options include the selection of the factorization rank (*k*), the number of iterations to perform before testing for convergence, the maximum number of iterations in case data never converge to a stable solution, and the stopping threshold for a convergence test. The test of convergence computes the assignment of samples to each factor, which it is represented in matrix **H** by the column index of the maximum value for each row. This sample classification is then compared with the one computed on the previous convergence test. If no differences are found after a certain number of consecutive tests (set by the stopping threshold parameter mentioned above), the algorithm is considered to have converged to a stable solution.

Finally, the output consists on two files containing the **W** and **H** matrices. The format of these files can be either a tab-separated text, or binary.

For instance, the following command:

***./*** NMF_GPU matrix.txt -k 4 -j 10 -t 40 -i 2000

processes the input file *“* matrix.txt *”* with a factorization rank of ***4***. The test of convergence will be performed each ***10*** iterations. If there are no relative differences in matrix **H** after ***40*** consecutive convergence tests, the algorithm has converged. Otherwise, a maximum of ***2000*** iterations will be performed. Finally, output matrices **W** and **H** are saved in files “matrix.txt_W.txt *”* and “matrix.txt_H.txt”, respectively.

The multi-GPU version works similarly. The executable file is named “NMF-mGPU”; however, the MPI standard requires the software to be executed through the mpiexec or mpirun commands.

Although the actual arguments may be MPI-implementation specific, a typical invocation command in a two-GPUs cluster, using similar parameters as the example above, should be the following:

Mpiexec -np 2 NMF_mGPU matrix.txt -k 4 -j 10 -t 40 -i 2000

The resulting output files are also similar as in the single-GPU version.

### Use case

We have analyzed some biological data as an example on how to use the tool. We have chosen data from [61], whose authors compared microarray expression data from samples of normal human Schwann cells (NHSC), dermal and plexiform NF1-derived primary benign neurofibroma Schwann cells (dNFSCs and pNFSCs), and malignant peripheral nerve sheath tumor cell lines (MPNST). In that work, they found that most MPNST cell lines share a transcriptional signature, which is different from NHSC cells. In addition, the set consisting on dNFSCs and pNFSCs also differs from the other cell types. Nevertheless, there are not clearly visible differences between such dermal and plexiform NFSCs.

We retrieved the expression data from Gene Expression Omnibus [52] (accession number GSE14038) in order to generate a data matrix where samples are in columns and gene probes are in rows. This was done using the GEOquery tool [62]. We then carried out two different analyses using *NMF-mGPU*.

**Figure 6:**
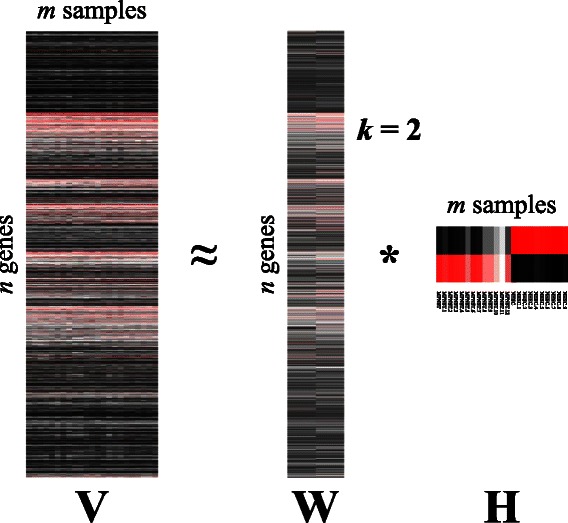
**Heatmap representation of the NMF applied to MPNST and NHSC cells.** Each matrix is represented as a heatmap, with each item representing a normalized expression value. Black color corresponds to zero (no expression values), while red items correspond to the maximum value. Sample names are reported in Matrix **H**.

For our first test, we generated a sub-matrix with only data from MPNST and NHSC cells, and executed our tool using the default parameters, including a factorization rank of two. We obtained two matrices: **H**, with metagene expression levels in each row; and **W**, whose columns represent the coefficients of each gene in that metagene. This can be considered a biclustering analysis due to the scatter nature of the NMF factorization [19,20]. Heatmap representations of all three matrices are shown in Figure 6. There is a clear distinction between the MPNST and the NHSC groups, both represented in matrix **H** as different expression levels of each metagene in the samples.

**Figure 7:**
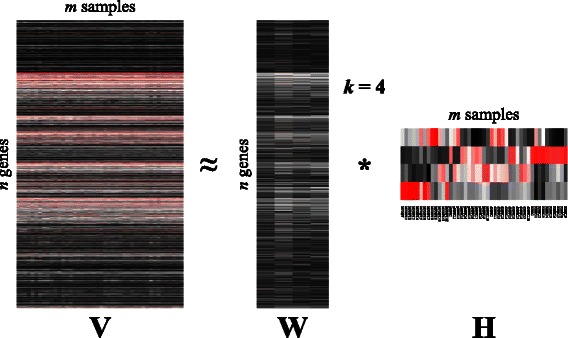
**Heatmap representation of the NMF applied to MPNST, dNFSCs, pNFSCs, and NHSC cells.** Each matrix is represented as a heatmap, with each item representing a normalized expression value. Black color corresponds to zero (no expression values), while red items correspond to the maximum value. Sample names are reported in Matrix **H**.

The second analysis corresponds to a sample classification of the four cell groups. We executed our tool using a factorization rank of four. The output matrices led us to the same conclusions as the authors of the study, where they identified two clearly differentiated groups, MPNST and NHSC, and two other classes containing a mix of the remaining samples with elements from dNFSCs and pNFSCs. Figure 7 depicts these findings, which are represented by the different metagene expression levels in matrix **H**.

Both analyses took just a few seconds in an old NVIDIA GeForce 8800 GTX (128 cores and 768 MB of memory), connected to a personal computer. Then, all heatmaps were generated using the *aheatmap* tool from the package *NMF* for R [25].

## Conclusions

The applications of GPUs in Bioinformatics are getting more and more attention due to their spectacular speedup improvements when compared to traditional CPU computations. Conventional high-performance clusters are still useful in many areas, but their cost is several orders higher than GPU devices, which are, in addition, already present on many modern personal computers. Furthermore, their greater acceptance has allowed commercial developments of multi-GPU systems that facilitate the exploitation of multiple levels of parallelization as discussed in this work. In life sciences, this represents an excellent opportunity to facilitate the daily work of bioinformaticians that are trying to extract biological meaning out of hundreds of gigabytes of experimental information. However, the spread of the use of this technology is still limited to those with programming experience on this type of devices. In this work, we wanted to make a very simple use case of a widely used algorithm, the NMF, in a GPU platform. Our main goal is to reach and help the final experimentalist in using this technology, either in a simple laptop or in a high-end GPU workstation. We hope this implementation helps in providing the Bioinformatics community with a way in presenting the GPU applications and we also hope that NMF algorithms can now be used in more complex problems with the help of the great performance provided by one or more GPU devices. As future work, we are planning to migrate the code to OpenCL, so it will be able to execute this application on GPU devices from other manufacturers (e.g., Intel, ATI, etc.).

## Availability and requirements

**Project name:** ***NMF-mGPU***

**Home page:** https://github.com/bioinfo-cnb/bionmf-gpu

**Operating system(s):** Mac OS X and Linux

**Programming language:** C/CUDA

**Other requirements:** CUDA Toolkit 4.2 or higher. The multi-GPU version requires, in addition, an MPI-2.0 library (e.g., Open-MPI, Mpich, etc.).

Please note that since CUDA 6.0, support for *old* devices (e.g., compute capability 1.0) and *old* operating systems (e.g., any 32-bits Linux, Mac OS X 10.7 Lion and below) has been removed. In that case, we recommend installing CUDA 5.5.

**Software License:** GPLv3+

**Any restrictions to use by non-academics:** none

### Consent and ethical approval

All datasets used for this article come from third-party works, whose authors had already made them publicly available on recognized scientific databases or institutional websites. These works, including any accession number, are properly cited in the manuscript.
